# Mismatch between global patient blood management policy and nursing education: evidence from four countries

**DOI:** 10.3389/fpubh.2026.1858749

**Published:** 2026-06-18

**Authors:** Jan Domaradzki, Piotr Jabkowski, Justyna Czekajewska, Marcin Piotr Walkowiak, Natalia Markwitz-Grzyb, Einat Doron, Reinhold Wolke, Petra Reiber, Akram Sanagoo, Leila Jouybari, Reza Jahanshahi, Zhanar Dostanova, Lyudmila Yermukhanova, Alua Miraleyeva, Dariusz Walkowiak

**Affiliations:** 1 Department of Social Sciences and Humanities, Poznan University of Medical Sciences, Poznan, Poland; 2 Faculty of Sociology, Adam Mickiewicz University, Poznan, Poland; 3 Department of Preventive Medicine, Adam Mickiewicz University, Poznan, Poland; 4 Independent Researcher, Binyamina, Israel; 5 Faculty for Social Sciences, Education and Nursing, University of Applied Science, Esslingen, Germany; 6 Faculty of Nursing and Midwifery, Golestan University of Medical Sciences, Gorgan, Iran; 7 Department of Public Health and Healthcare, West Kazakhstan Marat Ospanov Medical University, Aktobe, Kazakhstan; 8 Department of Psychology, West Kazakhstan Marat Ospanov Medical University, Aktobe, Kazakhstan; 9 Department of Organization and Management in Health Care, Poznan University of Medical Sciences, Poznan, Poland

**Keywords:** blood transfusions, bloodless medicine, medical education, non-blood management techniques, nursing education curriculum, patient blood management

## Abstract

**Background:**

Patient Blood Management (PBM) is an evidence-based approach to optimising transfusion practices and improving patient outcomes, yet its integration into nursing education remains limited and insufficiently explored across healthcare systems. This study examined nursing master’s students’ awareness, knowledge, attitudes, and educational needs related to PBM and non-blood management in a cross-national context.

**Methods:**

A cross-sectional web-based survey was conducted among 645 nursing master’s students from Poland, Germany, Kazakhstan, and Iran between October 2023 and April 2024. A purpose-built questionnaire assessed self-reported awareness and objective knowledge across PBM domains. Knowledge indices were analysed using Poisson regression to identify associations with socio-demographic and professional factors.

**Results:**

Students across all countries demonstrated consistently low objective knowledge despite moderate self-reported awareness, indicating a clear awareness-knowledge gap. Mean scores for self-reported knowledge ranged from 1.40 to 2.03, whereas mean objective knowledge scores ranged from 2.83 to 6.86 for non-blood management techniques, 1.91 to 5.53 for pharmacological PBM strategies, and 1.22 to 4.28 for PBM-related risks. Deficits were evident across key PBM domains, including alternative treatments, pharmacological strategies, and associated risks. Although some between-country variation was observed, overall patterns remained consistent. Higher knowledge levels were associated with greater professional experience, prior exposure to patients refusing transfusion, and selected socio-demographic factors. Most participants reported insufficient formal PBM education, with only a minority feeling adequately prepared, alongside a strong demand for structured training.

**Conclusion:**

PBM remains insufficiently embedded in nursing education, which may limit preparedness for safe and evidence-based transfusion practice. Strengthening its integration into curricula and postgraduate training may help bridge the gap between global PBM recommendations and clinical competence.

## 1 Introduction

Blood transfusion remains a cornerstone of modern medicine, widely used in surgical, oncological, and emergency care. However, it is also associated with clinically significant risks, including adverse reactions, immunological complications, and transmission of infections (1–5). At the same time, many healthcare systems face persistent challenges related to blood shortages, driven by demographic changes, ageing populations, and disruptions such as the COVID-19 pandemic (6–10).

In response to these challenges, Patient Blood Management (PBM) has emerged as a comprehensive, evidence-based approach aimed at optimising the use of blood products and improving patient outcomes (11–15). PBM encompasses strategies such as the management of preoperative anaemia, minimisation of perioperative blood loss, and the use of pharmacological and technological alternatives to transfusion (13, 16, 17). These interventions have been shown to reduce transfusion rates, healthcare costs, improve postoperative recovery, and enhance patient safety (13, 18–25).

In light of this evidence, the World Health Organisation has identified PBM as a global health priority and called for its integration into healthcare systems and professional education. In this study, “awareness” is used as an overarching concept encompassing both self-reported familiarity and objective knowledge of PBM-related practices (14, 15).

Despite increasing international recognition of PBM, its implementation remains uneven across countries and clinical settings. One of the key barriers is the insufficient preparation of healthcare professionals, particularly nurses, who play a central role in perioperative care, patient monitoring, and clinical decision-making. Existing studies suggest that knowledge of PBM and non-blood management techniques among nurses is often limited, which may compromise both patient safety and the effective implementation of evidence-based practices (26, 27). Previous studies among undergraduate healthcare students have also demonstrated variable knowledge and attitudes toward blood donation and transfusion practices, underscoring persistent educational gaps in trainee populations (28). This may reflect both variability in curriculum content and differences in the extent to which students are involved in clinical decision-making during training.

Importantly, most research to date has focused on practising clinicians within single-country contexts, with limited attention given to students entering the profession. As future healthcare providers, nursing students represent a critical group for understanding how well PBM principles are embedded within educational systems. Master’s-level nursing students represent a critical group, as they are expected to transition rapidly into advanced clinical roles requiring decision-making and evidence-based practice (29–31). Moreover, cross-national comparisons remain scarce, despite the global nature of PBM recommendations and the diversity of healthcare systems in which they are implemented. This study addresses this gap by examining the awareness, attitudes, and educational needs related to PBM and non-blood management techniques among nursing master’s students in Poland, Germany, Kazakhstan, and Iran. By comparing countries with differing healthcare infrastructures, cultural contexts, and educational systems, this study aims to identify common patterns and structural gaps in PBM education.

Specifically, the study seeks to answer the following research questions:

1. What is the level of knowledge of non-blood management techniques among nursing students?
2. What are their attitudes towards these approaches?
3. What are their educational needs in this area?
4. Which socio-demographic and professional factors are associated with their awareness?

By identifying systemic gaps in knowledge and training, this study contributes to the ongoing discussion on how to align nursing education with global PBM strategies and improve the quality and safety of patient care.

## 2 Materials and methods

### 2.1 Study design

This cross-sectional study was conducted as part of a broader international research initiative examining healthcare professionals’ knowledge, attitudes, and educational needs related to PBM and non-blood management strategies across different clinical and educational contexts (26). The present analysis focuses specifically on nursing master’s students. The study followed the Strengthening the Reporting of Observational Studies in Epidemiology (STROBE) guidelines (32).

### 2.2 Ethical issues

The study was conducted in accordance with the Declaration of Helsinki and relevant data protection regulations (33). Ethical approval was obtained from the Poznan University of Medical Sciences Bioethics Committee (KB – 760/22). Furthermore, ethical approval was also obtained from the ethical board of the West Kazakhstan Marat Ospanov Medical University and Kazakhstan and Golestan University of Medical Sciences, as well as the management board of the Master’s program of Esslingen University of Applied Sciences. Participation was voluntary and anonymous, and all participants signed an online informed written consent form to participate before completing the survey. No personal identifiers were collected.

### 2.3 Participants and setting

The study was conducted among nursing master’s students enrolled at four academic institutions: Poznan University of Medical Sciences (Poland), Esslingen University of Applied Sciences (Germany), West Kazakhstan Marat Ospanov Medical University and Kazakhstan (Kazakhstan), and Golestan University of Medical Sciences (Iran).

Participants were recruited using a convenience sampling approach during scheduled academic activities. Inclusion criteria were: 1. enrolment in a nursing master’s programme, 2. affiliation with one of the participating institutions, 3. informed consent, and 4. ability to complete an online questionnaire.

Master’s students were selected because, having completed a bachelor’s degree in nursing and obtained professional nursing qualifications, they are often clinically active during postgraduate education, increasing the likelihood of exposure to situations involving non-blood management techniques (29, 34, 35).

Although recruitment was standardised across sites, the final sample sizes differed substantially between countries, reflecting variations in cohort sizes and participation rates. This imbalance was accounted for in the interpretation of results and explicitly considered as a study limitation.

Participants were invited during regular academic sessions and accessed the questionnaire via a QR code or a direct link provided by local study coordinators. Participation was voluntary and not linked to academic evaluation or course requirements. To minimise potential coercion and power imbalance in the classroom setting, the questionnaire was completed individually and anonymously using an online platform without direct supervision or monitoring of responses. No financial or material incentives were offered for participation.

### 2.4 Instrument development

As no standardised instrument was available to assess knowledge and awareness of PBM and non-blood management techniques among nursing students, a structured questionnaire was developed specifically for this study. The development process followed established recommendations for survey design (36). First, a targeted literature review was conducted to identify key domains related to PBM, including knowledge of transfusion alternatives, pharmacological strategies, and associated risks. Based on this review, an initial item pool was generated (5, 13, 16–19, 21, 22, 24, 25, 37–39).

Content validity was established through expert evaluation by a multidisciplinary panel consisting of a physician, a nurse, a public health specialist, and a medical sociologist. The panel assessed the relevance, clarity, and completeness of items in relation to the study objectives. The preliminary version of the questionnaire was pilot-tested with a group of 20 nursing master’s students. Based on pilot feedback, minor revisions were introduced, including rewording of items and removal of ambiguous questions.

The final instrument consisted of 34 items across four domains: 1. general knowledge of transfusion and PBM, 2. knowledge of non-blood management techniques, 3. knowledge of pharmacological strategies, and 4. awareness of risks and complications (Supplementary file). Given the index-based structure of the questionnaire, in which items represent distinct aspects of knowledge rather than a single latent construct, internal consistency measures (e.g., Cronbach’s alpha) were not considered appropriate. Instead, emphasis was placed on content validity and expert-driven item selection. The instrument captures recognition-based knowledge rather than depth of understanding and should therefore be interpreted as an exploratory measure of awareness rather than a comprehensive assessment of competence.

### 2.5 Translation and cross-cultural adaptation

The questionnaire was originally developed in Polish and subsequently translated into English by two bilingual native speakers proficient in both languages. It was then adapted into German, Persian, Russian, and Kazakh using a forward-backwards translation procedure. Each language version was independently translated by bilingual experts and then back-translated to ensure conceptual equivalence. In each participating country, the translations and back-translations were independently performed by bilingual academics familiar with healthcare and nursing terminology. Minor contextual adaptations were made to ensure cultural relevance while preserving the original meaning of items. No formal assessment of measurement invariance across language versions was conducted; therefore, cross-country comparisons should be interpreted as exploratory and indicative of general patterns rather than strict equivalence.

### 2.6 Data collection

Data were collected between October 2023 and April 2024 using an anonymous web-based survey platform, and no personally identifiable information was recorded. Participants accessed the questionnaire via a QR code or a direct link provided during academic sessions. Before participation, all respondents were informed about the purpose of the study and provided electronic informed consent. The questionnaire required approximately 10–15 min to complete.

### 2.7 Variables and measurement

Knowledge of PBM and non-blood management techniques was assessed using four indices representing distinct dimensions: 1. self-reported knowledge, 2. knowledge of non-blood management techniques, 3. knowledge of pharmacological interventions, and 4. knowledge of associated risks.

Each index was constructed as a sum of dichotomous items, where a response was coded as 1 if correct and 0 if incorrect. This approach enabled the assessment of multiple independent domains of knowledge rather than a single aggregated construct. The survey participants could not skip the knowledge items and were asked to provide valid answers, as these items were central to the study.

### 2.8 Statistical analysis

All outcome variables were defined as count data and analysed using Poisson regression models. The logarithm of the expected value of each knowledge index was modelled as a linear combination of selected predictors. Independent variables included country, religious affiliation, professional seniority, and prior experience with patients refusing allogeneic blood transfusion. Countries were categorised as Germany (reference category), Iran, Kazakhstan, and Poland. Germany was selected as the reference category because it represents a well-established European healthcare system with relatively advanced transfusion medicine and PBM practices, facilitating interpretation of comparisons across different healthcare and educational contexts (40). Religious affiliation included atheist/agnostic (reference category), Catholic, other Christian, Muslim, and other religions. Professional seniority was categorised as none (reference category), up to 5 years, 6–10 years, and ≥11 years. Experience with transfusion refusal was coded as yes (reference category) or no.

To assess the robustness of findings, a stepwise modelling approach was applied. Model 1 included country as the sole predictor, whereas Model 2 additionally adjusted for all covariates. Given the unequal sample sizes across countries, results were interpreted with caution, with greater emphasis placed on the direction and consistency of effects than on precise cross-country comparisons. As all knowledge indices were defined as count outcomes, Poisson regression was selected as the main modelling approach. Overdispersion and overall model fit were considered during model specification. This approach was retained because incidence rate ratios provide a clear and interpretable summary of the associations between student characteristics and knowledge scores. However, alternative count models, such as negative binomial, quasi-Poisson, or zero-inflated models, were not formally compared in the present analysis. All analyses were conducted using R statistical software (version 4.3.1) (41).

## 3 Results

### 3.1 Study participants

In total, 645 nursing master’s students completed the questionnaire (Table 1). The response rates were as follows: Poland, 145 out of 188 (77.12%); Kazakhstan, 418 out of 462 (90.48%); Germany, 46 out of 58 (79.31%); and Iran, 36 out of 36 (100%). However, given that respondents from Kazakhstan constituted nearly two-thirds of the total sample, some overall descriptive patterns may have been predominantly driven by responses from the Kazakh subgroup. Students who refused to participate did so because they were either absent during the classes, lacked interest in the survey topic, or did not want to share their opinions.

**Table 1:** Study participants.

Characteristics	n	%	Mean	SD	Min	Max
Country						
Germany	46	7.1%				
Iran	36	5.6%				
Kazakhstan	418	64.8%				
Poland	145	22.5%				
Sex						
Female	592	91.8%				
Male	53	8.2%				
Seniority			12.2	12.3	0	45
Seniority—recoded						
None	42	6.5%				
Up to 5 years	260	40.3%				
6–10 years	91	14.1%				
11 + years	252	39.1%				
Confession						
Agnostic / atheist	73	11.3%				
Catholics	100	15.5%				
Other Christians	42	5.5%				
Muslims	283	43.9%				
Others	147	22.8%				
Having experience with a situation in which a person refused an allogeneic blood transfusion						
Yes	244	37.8%				
No	401	62.2%				

Since nursing studies are strongly gendered in all these countries, the sample consisted of 592 females (91.8%) and 53 males (8.2%). While most students were already professionally active (93.5%), 40.3% reported working as nurses for up to 5 years, 14.1% for 6 to 10 years, and 39.1% for more than 10 years. 37.8% of students declared having had previous experience with a patient who refused an allogeneic blood transfusion. 11.3% of respondents declared themselves as non-believers, either agnostics or atheists, while the majority identified as believers (88.7%). Of those, 43.9% identified themselves as Muslims, 15.3% as Catholics or other Christians, 5.5, and 22.8% declared other confessions.

### 3.2 Nursing master’s students’ awareness of non-blood management techniques and patient blood management

**Figure 1:**
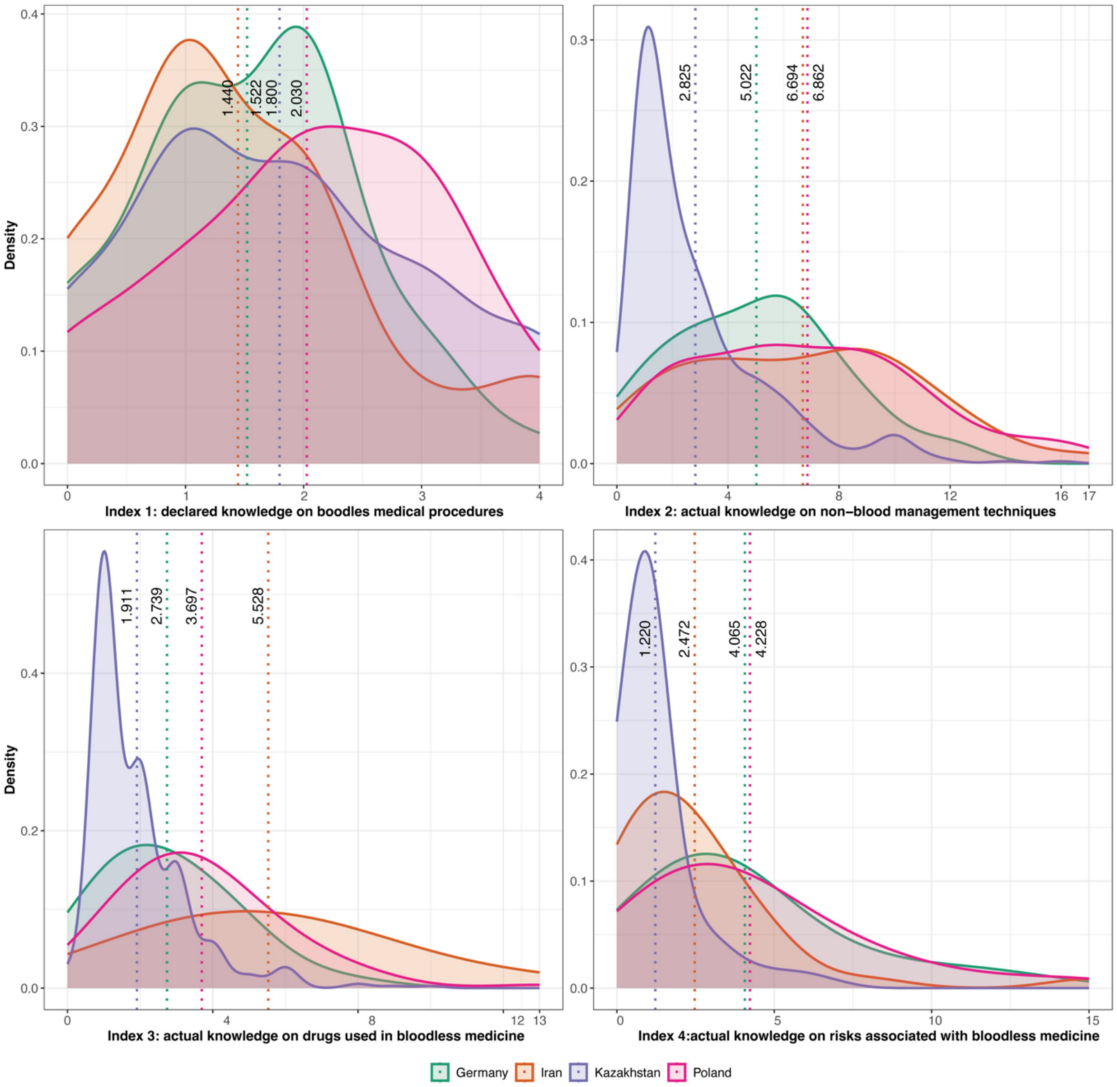
Density plots of the indexes of declared and actual knowledge.

Figure 1 shows a relatively similar distribution of scores across countries for Index 1, which measures self-reported knowledge, and notable differences for the remaining three indexes, which measure actual knowledge. For the actual knowledge indices, the scores observed in all four countries are skewed towards lower values (low actual knowledge score), with the distribution in Kazakhstan being extremely skewed (deficient actual knowledge). Detailed item-level descriptive statistics, including proportions of correct responses for individual knowledge items, are provided in the Supplementary materials.

When analysing the country’s means, it can be noticed that Polish nurses rated their knowledge at the highest average level for the index of declared knowledge (index 1) (mean = 2.03). In contrast, Iranian and German nurses rated their knowledge at the lowest level (means = 1.40 and 1.52, respectively), while Kazakh nurses’ self-assessment of their knowledge was rated as intermediate (mean = 1.80).

For the indices testing actual knowledge, the mean score for Kazakh nurses was always at the lowest level (Index 2 = 2.83; Index 3 = 1.91; Index 4 = 1.22). In contrast, Polish and Iranian nurses were at the top of the ranking defined by index 2 (actual knowledge of non-blood management techniques; means = 6.86 and 6.69, respectively). Iranian respondents were also at the top of the ranking defined by index 3 (knowledge of drugs used in PBM; mean = 5.53), and Polish and German nurses were at the top of the ranking defined by index 4 (knowledge of risks associated with PBM strategies; means = 4.28 and 4.07, respectively). Overall, mean scores for objective knowledge indices ranged from 2.83 to 6.86 for Index 2, from 1.91 to 5.53 for Index 3, and from 1.22 to 4.28 for Index 4 across countries. These descriptive patterns should be interpreted cautiously, as the pooled results may have been influenced by the predominance of respondents from Kazakhstan. We tested the statistical significance of these differences using regression analysis; however, the descriptive results presented in Figure 1 led us to expect more significant cross-country differences in the actual knowledge indices and less significant differences in the reported actual knowledge.

### 3.3 Findings from stepwise logistic regression analysis concerning factors impacting students’ awareness of non-blood management techniques and patient blood management

The Poisson regression analysis shows a significant association for some predictors of self-reported knowledge of non-blood management techniques and PBM principles (index 1) (Table 2). Poland shows a significant increase in reported knowledge compared to Germany, and these differences remain significant in Model 1.2, which includes additional variables (an increase of 33% in Model 1.1 and 60% in Model 1.2; small-to-moderate to moderate). In addition, seniority significantly increases the incidence rate (up to 59% higher for 11 + years; moderate). Conversely, having no experience with blood transfusion refusal significantly decreases the incidence rate (approx. 20% lower; small but statistically significant). Religious affiliation has no significant effect on the incidence rate.

**Table 2:** Summary of regression results: incidence rate ratios (IRR) regression parameters with their respective standard errors in brackets.

Regression parameters	Model 1.1	Model 1.2	Model 2.1	Model 2.2	Model 3.1	Model 3.2	Model 4.1	Model 4.2
	Index 1		Index 2		Index 3		Index 4	
Intercept	1.522^***^ (0.182)	1.096^*^ (0.229)	5.022^***^ (0.330)	4.338^***^ (0.532)	2.739^***^ (0.244)	2.254^***^ (0.363)	4.065^***^ (0.297)	4.169^***^ (0.620)
Iran [vs. Germany]	0.949 (0.174)	1.003 (0.210)	1.333^**^ (0.123)	1.718^***^ (0.202)	2.018^***^ (0.230)	2.349^***^ (0.344)	0.608^***^ (0.078)	0.746 (0.125)
Kazakhstan [vs. Germany]	1.181 (0.148)	1.315 (0.195)	0.563^***^ (0.040)	0.676^***^ (0.061)	0.698^***^ (0.067)	0.774^*^ (0.092)	0.300^***^ (0.026)	0.330^***^ (0.038)
Poland [vs. Germany]	1.332^*^ (0.177)	1.603^**^ (0.262)	1.366^***^ (0.100)	1.728^***^ (0.163)	1.350^**^ (0.134)	1.621^***^ (0.204)	1.040 (0.087)	0.981 (0.108)
Catholics [vs. Atheists/Agnostics]		0.987 (0.118)		0.875^*^ (0.057)		0.925 (0.083)		0.872 (0.071)
Other Christians [vs. Atheists/Agnostics]		1.093 (0.167)		1.024 (0.098)		1.092 (0.139)		0.726^*^ (0.092)
Muslims [vs. Atheists/Agnostics]		0.932 (0.114)		0.737^***^ (0.062)		0.886 (0.096)		0.668^***^ (0.079)
Others [vs. Atheists/Agnostics]		0.917 (0.110)		0.800^**^ (0.062)		0.928 (0.094)		0.714^**^ (0.074)
Seniority up to 5 years [vs. None]		1.477^**^ (0.213)		1.189^*^ (0.098)		1.139 (0.121)		1.111 (0.116)
Seniority 6 to 10 years [vs. None]		1.424^*^ (0.234)		1.232^*^ (0.124)		1.218 (0.153)		1.146 (0.148)
Seniority 11 + years [vs. None]		1.594^**^ (0.241)		1.505^***^ (0.138)		1.324^*^ (0.153)		1.281^*^ (0.155)
No experience with blood transfusion refusal [vs. Yes]		0.798^***^ (0.050)		0.838^***^ (0.036)		0.937 (0.051)		1.073 (0.066)
Observations	645	645	645	645	645	645	645	645
R2 Nagelkerke	0.022	0.088	0.551	0.606	0.409	0.430	0.581	0.598
AIC	2044.8	2033.2	3224.1	3166.8	2331.5	2332.9	2470.6	2465.1
log-Likelihood	−1018.4	−1004.6	−1608.1	−1571.4	−1161.7	−1154.4	−1231.3	−1220.6

* p-value *< 0*.05; ** *p-*value *<* 0.01; *** *p-*value < 0.001.

The regression results for actual knowledge of non-blood management techniques (Index 2) show that (in Model 2.1) Iran and Poland have significantly higher incidence rates than Germany, while Kazakhstan exhibits a significantly lower incidence rate (Iran/Poland 33–73% higher: small-to-moderate to moderate; Kazakhstan 24–44% lower: small-to-moderate). The cross-country differences remain significant after controlling for respondent characteristics. Religious affiliation significantly affects the incidence rate, with Catholics, Muslims, and others having lower incidence rates of actual knowledge compared to atheists and agnostics (reductions of 12–26%; small). Seniority also significantly increases the incidence rate, whereas having no experience of transfusion refusal significantly decreases the incidence rate for the outcome variable (seniority 19–51% higher: small-to-moderate; no-refusal experience 16–20% lower: small).

For actual knowledge of drugs used in non-blood management and PBM strategies (index 3), Model 3.1 shows that Iran and Poland have significantly higher incidence rates than Germany, while Kazakhstan shows a significantly lower incidence rate (Iran 2.0–2.35: large; Poland 35–62% higher: moderate; Kazakhstan 23–30% lower: small-to-moderate). Model 3.2, which includes additional variables, confirms the higher values for Iran and Poland, and the lower values for Kazakhstan. In addition, religious affiliation does not significantly affect the incidence rate, with Catholics, Muslims, and others showing no significant differences compared to atheists and agnostics. Seniority shows a significant increase only for those working as nurses for more than 11 years (32% higher; small-to-moderate). In addition, having no experience with patients refusing blood transfusions does not significantly affect the incidence rate.

The analysis identifies several significant predictors with notable cross-country differences for index 4. In Model 4.1, Iran and Kazakhstan show significantly lower incidence rates than Germany, whereas Poland shows no significant difference (Iran approx. 25–39% lower: small-to-moderate; Kazakhstan 67–70% lower: large). Model 4.2 confirms the lower incidence rates for Iran and Kazakhstan, with Poland still showing no significant difference. Religious affiliation has a significant effect on the incidence rate, with Muslims and others having lower incidence rates than atheists/agnostics, while Catholics show no significant difference (reductions of 29–33%: small-to-moderate). In addition, having no experience of transfusion refusal does not significantly affect the outcome variable, whereas seniority does (seniority approx. 28% higher for 11 + years: small-to-moderate).

### 3.4 Nursing master’s students’ educational needs

Nurses’ educational needs regarding non-blood management techniques differed significantly between countries (Table 3). Although 56.7% of all respondents believed that non-blood management techniques should be an integral part of medical care, it was primarily Polish and Iranian students who supported this claim (75.9 and 75%, respectively). In contrast, only 32.6% of German respondents agreed, and 54.3% were against the integration of these techniques into medical care. Kazakhstan had a balanced distribution with 51.2% in favour. The chi-square test confirmed significant differences between countries (χ^2^ = 81.75, df = 6, *p* < 0.001).

**Table 3:** Nurses’ educational needs on non-blood management techniques.

Question	Total	Germany	Iran	Kazakhstan	Poland
Should non-blood management techniques be an integral part of medical care?					
Yes	366 (56.7%)	15 (32.6%)	27 (75.0%)	214 (51.2%)	110 (75.9%)
No	108 (16.7%)	25 (54.3%)	4 (11.1%)	68 (16.3%)	11 (7.6%)
Do not know	171 (26.5%)	6 (13.0%)	5 (13.9%)	136 (32.5%)	24 (16.6%)
Chi-square test	-	81.75; df = 6; *p* < 0.001			
Did you have any classes on non-blood management techniques that involve strategies for avoiding blood transfusion and providing care to patients who refuse blood transfusion?					
Yes	173 (26.8%)	5 (10.9%)	2 (5.6%)	127 (30.4%)	39 (26.9%)
No	101 (15.7%)	0 (0.0%)	34 (94.4%)	0 (0.0%)	67 (46.2%)
Do not know	371 (57.5%)	41 (89.1%)	0 (0.0%)	291 (69.6%)	39 (26.9%)
Chi-square test	-	377.16; df = 6; *p* < 0.001			
Would you like to broaden your knowledge about non-blood management techniques?					
Yes	512 (79.4%)	17 (37.0%)	34 (94.4%)	336 (80.4%)	125 (86.2%)
No	67 (10.4%)	22 (47.8%)	1 (2.8%)	30 (7.2%)	14 (9.7%)
Do not know	66 (10.2%)	7 (15.2%)	1 (2.8%)	52 (12.4%)	6 (4.1%)
Chi-square test	-	90.99; df = 6; *p* < 0.001			
Do you think there should be a mandatory course on strategies to minimise blood loss during surgeries and prevent blood transfusion in medical curricula?					
Yes	510 (79.1%)	42 (91.3%)	30 (83.3%)	329 (78.7%)	109 (75.2%)
No	41 (6.4%)	1 (2.2%)	3 (8.3%)	24 (5.7%)	13 (9.0%)
Do not know	94 (14.6%)	3 (6.5%)	3 (8.3%)	65 (15.6%)	23 (15.9%)
Chi-square test	-	7.97; df = 6; *p* = 0.240			
Do you feel prepared for caring for a patient who requires treatment with non-blood management techniques?					
Yes	205 (31.8%)	1 (2.2%)	25 (69.4%)	153 (36.6%)	26 (17.9%)
No	275 (42.6%)	34 (73.9%)	8 (22.2%)	133 (31.8%)	100 (69.0%)
Do not know	165 (25.6%)	11 (23.9%)	3 (8.3%)	132 (31.6%)	19 (13.1%)
Chi-square test	-	108.63; df = 6; *p* < 0.001			

* p-value *< 0*.05; ** *p-*value *<* 0.01; *** *p-*value < 0.001.

While only 26.8% of all students declared having received formal training in non-blood management techniques, the lowest rate was observed among Iranian students (5.6%), and the highest among Kazakh and Polish students (30.4 and 26.9%, respectively). The chi-squared test showed significant differences (χ^2^ = 377.16, df = 6, *p* < 0.001).

79.4% of all respondents expressed the wish to increase their knowledge of non-blood management techniques. This support was the highest in Iran (94.4%) and Poland (86.2%), followed by Kazakh (80.4%). In contrast, only 37% of German nurses were interested in broadening their knowledge, and 47.8% expressed no such interest. The chi-square test confirmed these differences as significant (χ^2^ = 90.99, df = 6, *p* < 0.001).

Simultaneously, 79.1% of students believed that there should be a mandatory course on strategies to minimise blood loss and prevent blood transfusion in medical curricula. German respondents were the most supportive (91.3%), followed by Iranian students (83.3%), Kazakh (78.7%), and Polish (75.2%). The chi-square test showed no significant differences (χ^2^ = 7.97, df = 6, *p* > 0.05).

Overall, less than one-third of students felt prepared to care for patients requiring non-blood management techniques (31.8%). However, there were significant differences between countries. Iranian students felt most prepared (69.4%), followed by Kazakhstan (36.6%). In contrast, most German (73.9%) and Polish (69.0%) respondents felt unprepared. This pattern suggests lower levels of perceived preparedness among students in Germany and Poland. The chi-square test revealed significant differences (χ^2^ = 108.63, df = 6, *p* < 0.001).

## 4 Discussion

This study provides cross-national evidence of a consistent and substantial gap in nursing students’ knowledge of PBM and non-blood management techniques. Importantly, this deficit was observed across all four countries, despite differences in healthcare systems, cultural contexts, and educational structures (26, 27). These findings suggest that limited awareness of PBM is not a local or context-specific issue, but rather reflects a broader structural gap in nursing education. Importantly, the knowledge indices used in this study reflect recognition-based awareness rather than comprehensive clinical competence, and should therefore be interpreted as indicative rather than definitive measures of PBM literacy.

The results reveal a notable mismatch between global health policy and professional training. Although the World Health Organisation has widely endorsed PBM as a standard of care (14, 15), its principles do not appear to be systematically integrated into nursing curricula. This disconnect raises concerns about the preparedness of future nurses to implement evidence-based transfusion practices and to participate effectively in multidisciplinary PBM programs. The observed cross-country variation further suggests that PBM-related content may be inconsistently integrated across nursing curricula and clinical training environments. A particularly important finding is the discrepancy between self-assessed and actual knowledge. Similar discrepancies between perceived and objective knowledge have been reported in other areas of nursing education, including organ donation and blood-related practices (28, 42–45), suggesting a broader pattern in professional training (26, 46). Consistent with findings reported by Babker et al. (28) among undergraduate healthcare students in the United Arab Emirates, our results similarly indicate a gap between self-reported awareness and objective knowledge of blood-related practices. This pattern suggests the presence of overconfidence or superficial familiarity with the topic, which may further hinder the safe application of PBM principles in clinical practice.

The study also highlights the role of experiential and contextual factors in shaping knowledge. Greater professional seniority and prior exposure to patients refusing transfusion were consistently associated with higher levels of awareness. This suggests that practical experience may currently play a greater role than formal education in shaping PBM-related awareness. At the same time, the observed associations with religious affiliation point to the influence of cultural and ethical contexts, particularly in relation to transfusion refusal (26, 46–48). However, these effects varied across domains of knowledge, suggesting that they should be interpreted cautiously and within broader socio-cultural frameworks. These associations should not be interpreted as causal or culturally deterministic, but rather as indicative of broader contextual influences that were not directly measured in this study.

Although the general pattern of limited PBM knowledge was observed across all participating countries, some cross-country differences should be interpreted in relation to differences in educational systems, clinical exposure, healthcare organisation, and broader cultural contexts. For example, the relatively higher scores observed among Polish and Iranian students in selected domains may reflect differences in curricular emphasis, clinical training intensity, or greater exposure to ethical and transfusion-related dilemmas during education. Previous research from Poland suggests that healthcare professionals often report limited preparedness for PBM and non-blood management, while prior exposure to transfusion refusal and transcultural care may nevertheless shape attitudes and awareness regarding PBM-related issues (26, 49, 50). In the Polish context, this may partially reflect greater educational and clinical exposure to transfusion refusal among Jehovah’s Witnesses, a relatively visible religious minority frequently discussed in transcultural nursing and medical education. In contrast, the comparatively higher scores observed among German students may reflect the longer-standing implementation of PBM principles, stronger integration of transfusion medicine within the healthcare system, and greater emphasis on ethical reflection, patient autonomy, and physician responsibility in blood management decisions (51). Previous international research has similarly demonstrated that knowledge of PBM and bloodless medicine is strongly shaped by institutional education, clinical training opportunities, and healthcare system organisation (23, 27). Recent literature has further emphasised that successful PBM implementation depends not only on clinical protocols but also on interdisciplinary education, ethical awareness, institutional support for patient-centred decision-making, and adequate transfusion medicine training across healthcare systems (52). The consistently lower scores observed among Kazakh students may also reflect differences in the pace of PBM implementation, access to specialised educational resources, or opportunities for clinical exposure within evolving healthcare and nursing education systems. However, these interpretations remain speculative, as the present study did not directly assess curricular content, institutional differences, or national educational standards. Therefore, caution is warranted in attributing the observed differences solely to national, cultural, or educational characteristics. Moreover, because respondents from Kazakhstan constituted the majority of the study sample, some overall trends may have been disproportionately shaped by responses from this subgroup.

Taken together, these findings point to a systemic issue in the organisation of nursing education. PBM appears to be insufficiently or inconsistently embedded in formal curricula and is often treated as a specialised or physician-driven domain (19, 38). As a result, nurses may be underprepared to engage in key aspects of transfusion management, including patient education, risk assessment, and participation in PBM strategies (27). These findings may reflect a broader pattern in healthcare training, where emerging interdisciplinary approaches are not fully translated into educational practice.

From a policy perspective, the findings underscore the need to align nursing education with global PBM strategies. Integrating PBM into undergraduate and postgraduate curricula, as well as continuing professional development, could help bridge the gap between evidence-based recommendations and clinical implementation. In addition, incorporating case-based learning, simulation, and interprofessional training may enhance both competence and confidence in managing patients who require or refuse transfusion (53).

Finally, the cross-national design of this study highlights that the observed educational gaps persist across diverse healthcare systems. In addition, variation in response rates across countries, particularly the exceptionally high participation observed in one country (Iran), may reflect classroom-based recruitment and should therefore be interpreted with caution due to potential social desirability or perceived voluntariness effects. This suggests that addressing them will require not only local curriculum reforms but also broader international efforts to standardise PBM education and promote its integration into healthcare training frameworks. Addressing this gap may require not only curriculum revision but also closer integration between academic education and clinical practice environments.

### 4.1 Strengths and limitations

Several limitations of this study should be acknowledged. First, the use of a convenience sampling strategy and the inclusion of a single institution per country limit the generalisability of the findings. The sample may not fully represent all nursing master’s students within the respective national contexts. In addition, recruitment procedures based on classroom invitations and QR-code access to the online survey may have contributed to selection bias by favouring participation among students who were more academically engaged or more regularly present during classes. Furthermore, the sample was predominantly female (91.8%), which may limit the transferability of the findings to male nurses, although this distribution reflects the gender structure of the nursing profession. Second, the sample sizes differed substantially between countries, with a predominance of participants from Kazakhstan. This imbalance partly reflects structural differences in nursing education systems, including variations in cohort size, programme organisation, and accessibility of master’s-level training across countries. This heterogeneity itself may be considered an important contextual factor, reflecting broader differences in the organisation of nursing education across healthcare systems. While these differences are inherent to cross-national research, they may affect comparability. Therefore, the results should be interpreted with caution, with emphasis placed on overall patterns rather than direct national comparisons. Given the predominance of respondents from Kazakhstan, some overall patterns observed in the pooled analyses may have been disproportionately influenced by responses from this group. Accordingly, cross-country comparisons should be interpreted as exploratory rather than definitive. No additional sensitivity analyses (e.g., weighting or bootstrapping) were conducted, which may further limit the robustness of cross-country comparisons. In addition, although Poisson regression was selected for its interpretability and suitability for count data, alternative count-based modelling approaches were not formally compared, which may limit the assessment of model robustness. Third, the study relied on a self-developed questionnaire. While content validity was ensured through expert review and pilot testing, the instrument was not formally psychometrically validated. Moreover, although the questionnaire underwent translation and verification procedures, the use of multiple language versions may have introduced subtle differences in interpretation across countries, potentially affecting response comparability. However, given the absence of standardised tools in this field, the use of a purpose-built instrument was considered appropriate for exploratory analysis. Fourth, due to the cross-sectional design, causal relationships between variables cannot be inferred. The associations identified should be interpreted as correlational rather than causal. Finally, although participation was voluntary and anonymous, data collection took place in academic settings, which may have introduced a degree of response bias. In particular, the exceptionally high response rate observed in some countries should be interpreted in light of contextual and organisational factors. Despite these limitations, the study provides valuable cross-national insights into an underexplored area and highlights consistent patterns that warrant further investigation.

## 5 Conclusion

This study demonstrates that nursing master’s students across four diverse countries exhibit consistently low levels of knowledge regarding PBM and non-blood management techniques. These deficits were observed irrespective of national context, suggesting that they reflect a broader structural gap rather than isolated educational shortcomings. The findings reveal a clear mismatch between global health policy and professional training. Although PBM is widely promoted as a standard of care, its principles are not sufficiently embedded in nursing education. As a result, future nurses may enter clinical practice without the competencies required to support evidence-based transfusion management and patient-centred care.

Importantly, the study highlights that practical experience, rather than formal education, remains the primary source of PBM-related knowledge. This underscores the need for more systematic and structured educational approaches. Addressing these gaps will require the integration of dedicated PBM modules, blood conservation methods, and transfusion-free clinical strategies into undergraduate and postgraduate nursing curricula. Educational programmes should include topics such as preoperative anaemia management, transfusion-related risks, pharmacological and non-blood management strategies, and ethical aspects of informed consent and transfusion refusal. Practical educational components, including case-based learning, simulation exercises, and interprofessional workshops, may further strengthen clinical competence and ethical decision-making. In addition, PBM principles should be embedded in continuing professional development programmes to ensure that qualified nurses remain up to date with evolving transfusion-free practices and international recommendations. Integrating academic curricula with national and international PBM policies may help promote consistent evidence-based practice, improve patient safety, optimise resource use, and strengthen the role of nurses in multidisciplinary care.

## Data Availability

The raw data supporting the conclusions of this article will be made available by the authors, without undue reservation.
