# Supplementary material for: Mismatch between global patient blood management policy and nursing education: evidence from four countries
Source: Front Public Health. 2026 Jun 18;14:1858749. doi: 10.3389/fpubh.2026.1858749 (PMC13323678; doi:10.3389/fpubh.2026.1858749)
Supplement: Supplementary file 1 [file Data_Sheet_1.PDF]

Attitudes of nursing students toward bloodless medicine

Dear Student!

My name is Jan Domaradzki, and I work at Poznan University of Medical Sciences, Poznan, Poland. Together with my research team, we are conducting a project to understand better nursing students' knowledge and attitudes towards bloodless medicine, i.e., medical care that does not use blood products, including transfusions. We invite you to share your opinion on this important topic.

This survey should only take up to 7-10 minutes of your time. At the same time, we assure you that this survey is entirely anonymous and confidential. As all responses are anonymous, they cannot be traced back to the respondent. Moreover, while no personally identifiable information is captured, your responses will be combined with those of many others and summarised in a report to protect your anonymity further. All information gathered will be used only for scientific purposes.

Your feedback is very important and will help our University improve our work.

For any questions or if you need assistance to complete this questionnaire, please contact:

Dr hab. Jan Domaradzki, prof. UMP
e-mail: jandomar@ump.edu.pl
Mobile: +48 695 324 630

We appreciate your time and help.

Warm regards

Jan Domaradzki

CONSENT FORM:

1. The project Manager informed me about the planned scientific research, particularly its assumptions, goals, course, and research method.
2. I understand all the information regarding this research study.
3. I have been informed that participation in the research study is entirely voluntary.
4. I have been informed that I can withdraw from participation in this research study without giving reasons, and my decision will not result in any penalties.
5. I have been informed that I can contact the persons indicated in the information if I have any questions or doubts during the research study.
6. I voluntarily consent to participate in the study.

☐ yes
☐ no

Part 1. In this part, we ask about your knowledge of and attitudes toward bloodless medicine:

Is there any alternative to blood transfusion?
 yes
 no

Can blood transfusions be avoided even during significant surgeries?
 yes
 no

Have you ever heard of bloodless medicine?
 yes
 no

What is bloodless medicine?
 medical care that does not use blood
 medical care that uses only autologous blood donation
 medical care that uses only allogeneic blood
 I do not know

Are you familiar with any of the alternatives to blood transfusion? ***Please mark all responses you find correct:***
 Extra-Corporeal Membrane Oxygenation (ECMO)
 artificial kidney (dialyser)
 plasma expanders
 blood volume expanders to prevent hypovolemic shock
 using non-blood fluids
 haemostatic dressings
 cellulose-based wound dressings
 fibrin glues
 giving iron supplements
 administered intravenous haemostatic agents (i.e., tranexamic acid)
 plasma-derived clotting factor concentrates (i.e., fibrinogen or factors) administered to people with haemophilia
 preoperative blood clotting activation
 hematopoietic growth factors (i.e. erythropoietin)
 blood recovery: blood filtering and returning the filtered blood to the patient
 extracorporeal circulation machines
 the use of the haemostatic scalpel (i.e. electrocautery, ultrasonic scalpel, laser)
 minimally invasive surgery (laparoscopy, surgical robotics)
 others

What types of drugs are being used in bloodless medicine? ***Please mark all responses you find correct:***
 Ringer's lactate
 iron supplements
 saline solution
 dextran
 gelatine (i.e., Haemaccel)
 erythropoietin
 hydroxyethyl starch (HES)
 romiplostim, eltrombopag, interleukin-11
 GM-CSF, G-CSF
 aprotinin
 antifibrinolytic drugs
 desmopressin
 dietary supplements
 others

Is bloodless medicine associated with risk or complications?
 yes
 no
 I do not know

What types of risks and complications are associated with bloodless medicine? ***Please mark all responses you find correct:***
 there are no complications related to bloodless medicine
 fever
 hypertension
 allergic skin reactions, i.e. erythema, itching
 vomiting
 dark colour of urine
 cardiovascular and respiratory disorders
 decrease in haemoglobin
 thrombosis
 bleeding and symptoms of haemorrhagic diathesis
 anaphylactic shock
 transfusion-related acute lung injury
 sepsis
 bacterial and viral complications
 hepatitis
 death
 others

Does the World Health Organisation recommend using strategies to optimise blood usage for patient health to avoid blood transfusion?
 yes
 no

Should non-blood management techniques be an integral part of medical care?
 definitely yes
 rather yes
 rather no
 definitely no
 I do not know

Did you have any classes on non-blood management techniques (bloodless medicine) that involve strategies for avoiding blood transfusion and providing care to patients who refuse blood transfusion?
 yes
 no

Would you like to broaden your knowledge about non-blood management techniques?
 definitely yes
 rather yes
 rather no
 definitely no
 I do not know

Should there be a mandatory course on strategies to minimise blood loss during surgeries and prevent blood transfusion (patient blood management and non-blood management techniques) in medical curricula?
 definitely yes
 rather yes
 rather no
 definitely no
 I do not know

Do you feel prepared for caring for a patient who requires treatment with non-blood management techniques?
 definitely yes
 rather yes
 rather no
 definitely no
 I do not know

How would you rate your knowledge of non-blood management techniques?
 very good
 fair enough
 insufficient
 very poor

Part 2. In this part, we ask about your demographic characteristics

Country
 Germany
 Iran
 Kazakhstan
 Poland

Sex
 female
 male

Year of Study
 4
 5
 6

Seniority (in years)
…………………

Confession
 Roman Catholicism
 Orthodox Christianity
 Lutheranism
 Calvinism (Presbyterians)
 Pentecostal
 Jehovah's
 Other Protestant
 Islam
 Judaism
 Secular theism
 Agnosticism
 Atheism

During your professional career, have you ever been in a situation where a person refused a blood transfusion?
 yes
 no
