# Supplementary material for: Mismatch between global patient blood management policy and nursing education: evidence from four countries
Source: Front Public Health. 2026 Jun 18;14:1858749. doi: 10.3389/fpubh.2026.1858749 (PMC13323678; doi:10.3389/fpubh.2026.1858749)
Supplement: Supplementary file 2 [file Data_Sheet_2.PDF]

Table 2. **General knowledge on bloodless medicine**

**Category**	**Have heard of bloodless medicine**	**Know what is bloodless medicine**	**Know that allogenic blood transfusion can be avoided**	**Know that WHO recommends optimising blood usage**
Total	0.371	0.452	0.657	0.716
Country				
Germany	0.304	0.500	0.565	0.848
Kazakhstan: Kazakhs	0.283	0.426	0.689	0.701
Kazakhstan: Russians	0.371	0.419	0.563	0.677
Poland	0.545	0.517	0.738	0.745
Seniority				
None	0.190	0.357	0.500	0.643
Up to 5 years	0.452	0.460	0.707	0.732
6-10 years	0.326	0.384	0.674	0.756
11+ years	0.339	0.483	0.628	0.698
Confession				
Agnostic / Atheist	0.479	0.438	0.781	0.781
Catholics	0.550	0.550	0.670	0.740
Other Christians	0.452	0.524	0.619	0.810
Muslims	0.287	0.381	0.644	0.741
Others	0.315	0.497	0.622	0.608
Role of religion in life				
Religious	0.342	0.432	0.642	0.733
Ambivalent / non-religious	0.391	0.464	0.667	0.705
Blood transfusion refusal - experienced before				
Yes	0.388	0.461	0.699	0.758
No	0.362	0.446	0.633	0.692

Table 3. **Specific knowledge of non-blood management techniques**

Statement	Overall fraction	Germany	Kazakhstan: Kazakhs	Kazakhstan: Russians	Poland
Knowledge of any alternative (bloodless) treatment methods:					
-- Yes	0.427	0.435	0.398	0.323	0.593
Familiarity with any of the alternatives to blood transfusion:					
-- Extra-Corporeal Membrane Oxygenation (ECMO)	0.389	0.130	0.275	0.377	0.683
-- Artificial kidney (dialyzer)	0.389	0.261	0.275	0.305	0.724
-- Plasma expanders	0.394	0.217	0.359	0.431	0.469
-- Blood volume expanders to prevent hypovolemic shock	0.309	0.304	0.179	0.251	0.600
-- Using non-blood fluids	0.255	0.500	0.195	0.132	0.421
-- Haemostatic dressings	0.200	0.109	0.092	0.246	0.366
-- Cellulose-based wound dressings	0.056	0.087	0.040	0.042	0.090
-- Fibrin glues	0.064	0.152	0.028	0.036	0.131
-- Giving iron supplements	0.376	0.696	0.295	0.269	0.538
-- Administered intravenous haemostatic agents	0.199	0.283	0.120	0.251	0.248
-- Plasma-derived clotting factor concentrates	0.250	0.435	0.163	0.192	0.407
-- Preoperative blood clotting activation	0.163	0.261	0.112	0.108	0.283
-- Hematopoietic growth factors	0.123	0.304	0.000	0.078	0.331
-- Blood filtering and returning the filtered blood	0.227	0.457	0.163	0.114	0.393
-- Extracorporeal circulation machines	0.118	0.109	0.048	0.078	0.290
-- The use of the haemostatic scalpel	0.182	0.217	0.084	0.162	0.366
-- Minimally invasive surgery	0.259	0.500	0.096	0.210	0.524
Knowledge of drugs being used in bloodless medicine					
-- Ringer's lactate	0.305	0.261	0.167	0.246	0.628
-- Iron supplements	0.609	0.783	0.629	0.497	0.648
-- Saline solution	0.365	0.674	0.199	0.389	0.524
-- Dextran	0.107	0.043	0.000	0.186	0.221
-- Gelatine (i.e. Haemaccel)	0.110	0.065	0.116	0.036	0.200
-- Erythropoietin	0.213	0.478	0.000	0.186	0.531
-- Hydroxyethyl starch (HES)	0.179	0.043	0.199	0.168	0.200
-- Romiplostim, eltrombopag, interleukin-11	0.080	0.043	0.116	0.060	0.055
-- GM-CSF, G-CSF	0.044	0.065	0.048	0.036	0.041
-- Aprotinin	0.034	0.022	0.000	0.042	0.090
-- Antifibrinolytic drugs	0.153	0.109	0.048	0.150	0.352
-- Desmopressin	0.044	0.022	0.028	0.030	0.097
-- Dietary supplements	0.154	0.130	0.219	0.102	0.110

Table 4. **Perception of specific risks and complications associated with a) blood transfusion and b) bloodless medicine**

Risk and complications	Overall fraction	Germany	Kazakhstan: Kazakhs	Kazakhstan: Russians	Poland
Types of risks and complications associated with blood transfusion:					
-- Fever	0.099	0.022	0.135	0.132	0.021
-- Hypertension	0.512	0.957	0.203	0.533	0.883
-- Allergic skin reactions, i.e. erythema, itching	0.506	0.761	0.339	0.341	0.903
-- Vomiting	0.189	0.370	0.092	0.120	0.379
-- Dark colour of urine	0.614	0.761	0.538	0.503	0.828
-- Cardiovascular and respiratory disorders	0.243	0.000	0.259	0.174	0.372
-- Decrease in haemoglobin	0.140	0.000	0.159	0.150	0.138
-- Thrombosis	0.373	0.000	0.271	0.323	0.724
-- Bleeding and symptoms of haemorrhagic diathesis	0.123	0.239	0.096	0.090	0.172
-- Anaphylactic shock	0.243	0.565	0.131	0.198	0.386
-- Sepsis	0.232	0.370	0.167	0.168	0.372
-- Bacterial and viral complications	0.516	0.739	0.410	0.425	0.731
-- Hepatitis	0.149	0.000	0.000	0.000	0.628
-- Death	0.335	0.717	0.175	0.299	0.531
Types of risks and complications associated with bloodless medicine:					
-- Fever	0.174	0.065	0.203	0.251	0.069
-- Hypertension	0.190	0.326	0.036	0.174	0.434
-- Allergic skin reactions, i.e. erythema, itching	0.122	0.326	0.016	0.132	0.228
-- Vomiting	0.286	0.370	0.143	0.365	0.414
-- Dark colour of urine	0.143	0.239	0.040	0.162	0.269
-- Cardiovascular and respiratory disorders	0.077	0.239	0.036	0.042	0.138
-- Decrease in haemoglobin	0.213	0.348	0.060	0.222	0.428
-- Thrombosis	0.223	0.435	0.088	0.162	0.462
-- Bleeding and symptoms of haemorrhagic diathesis	0.102	0.283	0.016	0.048	0.255
-- Anaphylactic shock	0.122	0.217	0.068	0.096	0.214
-- Sepsis	0.228	0.304	0.163	0.251	0.290
-- Bacterial and viral complications	0.053	0.000	0.016	0.000	0.193
-- Hepatitis	0.105	0.217	0.012	0.072	0.269
-- Death	0.110	0.217	0.044	0.060	0.248
